# Comparative Analysis of Growth and Physiological Responses of Sugarcane Elite Genotypes to Water Stress and Sandy Loam Soils

**DOI:** 10.3390/plants12152759

**Published:** 2023-07-25

**Authors:** Muhammad Sajid, Muhammad Amjid, Hassan Munir, Muhammad Ahmad, Usman Zulfiqar, Muhammad Fraz Ali, Mohammad Abul Farah, Mohamed A. A. Ahmed, Arkadiusz Artyszak

**Affiliations:** 1 Department of Agronomy, University of Agriculture, Faisalabad 38040, Pakistanamjidm70@gmail.com (M.A.); hmbajwauaf@gmail.com (H.M.); ahmadbajwa516@gmail.com (M.A.); 2 Department of Agronomy, Faculty of Agriculture and Environment, The Islamia University of Bahawalpur, Bahawalpur 63100, Pakistan; 3 College of Agronomy, Northwest A & F University, Xianyang 712100, China; frazali15@gmail.com; 4 Department of Zoology, College of Science, King Saud University, Riyadh 11451, Saudi Arabia; mfarah@ksu.edu.sa; 5 Plant Production Department (Horticulture—Medicinal and Aromatic Plants), Faculty of Agriculture (Saba Basha), Alexandria University, Alexandria 21531, Egypt; drmohamedmarey19@alexu.edu.eg; 6 School of Agriculture, Yunnan University, Kunming 650091, China; 7 Institute of Agriculture, Warsaw University of Life Sciences—SGGW, Nowoursynowska 159, 02-776 Warsaw, Poland; arkadiusz_artyszak@sggw.edu.pl

**Keywords:** sugarcane, genotypes, drought, physiology, sandy loam

## Abstract

Stumpy irrigation water availability is extremely important for sugarcane production in Pakistan today. This issue is rising inversely to river flow due to inadequate water distribution and an uneven rainfall pattern. Sugarcane growth faces a shortage of available water for plant uptake due to the low water–holding capacity of sandy loam soil, particularly under conventional flood irrigation methods. To address this problem, sugarcane clones were evaluated for their agronomic and physiological traits under conditions of low water availability in sandy loam soil. Ten cane genotypes, HSF–240, SPF–213, CPF–249, CP 77–400, S2008–FD–19, S2006–US–469, S2007–AUS–384, S2003–US–633, S2003–US–127, and S2006–US–658, were exposed to four levels of water deficit created through skip irrigations. These deficit levels occurred during the 9th, 11th, 13th, and 16th irrigations at alternate deficit levels between 2020 and 2022. Physiological data were collected during the tillering and grand growth stages (elongation) in response to the water deficit. The sugarcane clones S2006–US–658, S2007–AUS–384, and HSF–240 exhibited resistance to low water availability at both the tillering and grand growth stages. Following them, genotypes S2006–US–658, S2007–AUS–384, and HSF–240 performed better and were also found to be statistically significant. Clones susceptible to water deficit in terms of growth and development were identified as CP 77–400, S2008–FD–19, S2006–US–469, and S2003–US–633. These genotypes showed reduced photosynthetic rate, transpiration rate, stomatal conductance, relative water content, cane yield, and proline content under stressed conditions. Therefore, genotypes S2006–US–658, S2007–AUS–384, and HSF–240 were better performers concerning physiological traits under water deficit and sandy loam soil in both years. Moreover, a significant positive correlation was assessed between agronomic traits and photosynthetic rats. This study highlights that sugarcane can sustain its growth and development even with less irrigation frequency or moisture availability, albeit with certain specific variations.

## 1. Introduction

Sugarcane holds significant global importance, accounting for approximately 85% of the world’s sugar consumption. In recent years, there has been a rapid expansion of sugarcane planting for biofuel production, including the emergence of energy canes. Cultivating sugarcane is a vital endeavor worldwide due to its multifaceted impact on nutrition, the environment, society, and the economy. It offers the potential for productive diversification through co–products and by–products. With cultivation spanning over 105 countries, sugarcane has a long–standing history of enhancing socioeconomic conditions from its inception to its present–day production [1]. In recent years, there has been a notable expansion in sugarcane cultivation worldwide. However, data for 2019–2020 indicates a decline in global sugar production by approximately 3% to 1745 million tons. This decrease can be attributed to various factors, including a significant drop of 5 million tons in India’s production. The decline in India’s sugar production is primarily due to reduced cultivation area and lower expected yields. Despite this decline, Brazil and India remain in close competition as the top sugar–producing countries globally. Both countries make made significant contributions to global sugar production. Additionally, Pakistan holds the fifth position in terms of sugar production [2].

Drought is a critical threat to sugarcane production due to contagious water resources and uneven rainfall patterns in Pakistan. These factors limit the growth and development of sugarcane significantly [3]. Screening sugarcane clones is an authentic means for exploiting the genetic potential to a higher extent, particularly under stressful conditions, as adopted by Javed et al. [4]. Being a C_4_ specie, sugarcane has four distinct growth phases, germination, tillering, grand growth, and maturity, which significantly impact growth and ultimate yield statistics [5]. 

Drought stress adversely impacts the different phenological stages of tillering and elongation, critical phases where internodal length depends highly on water availability and vice versa [6,7]. Physiologically, such delays in the growth and development of plants are expressed by three mechanisms, i.e., reduced absorption of photo–synthetically active radiations due to limited canopy size, poor radiation use efficiency due to sun–leeward leaf orientation, and narrow sizing of leaf in response to soil moisture deficit [8]. Furthermore, numerous ongoing physiological processes under moderate and severe water–stressed conditions, such as photosynthesis, transpiration, and stomatal conductance, are hindered. Photosynthesis is comparatively more sensitive among several physiological processes, which slows the turgor–based rate of stomatal conductance [9]. The reduction in stomatal conductance to conserve water under severely stressed conditions couples with a decreased CO_2_ fixation rate in the diffusion site of bundle sheet cells in the surrounding areas of ribulose 1,5–bisphosphate carboxylase oxygenase (Rubisco), resulting in poor fresh weight accumulation in the sugarcane [10,11]. Moreover, leaf water status directly interacted with stomatal conductance in water–deficient environments. Hence, physiological traits such as water potential, leaf osmotic potential, and stomatal conductance have been used to assess the impact of water deficit on sugarcane, which can provide the basis for screening the sugarcane clones for drought tolerance [12]. Such physiological processes are interlinked with each other and expose possibilities of growth and development assurance under water–scarce conditions [13]. Sugarcane’s aptitude for supporting various physiological processes during water stress leads to understanding its genetic flexibility for growth and yield performance in water scarcity regions [11,13]. Many other physiological traits, such as stomatal conductance, relative water contents, leaf osmotic potential, leaf temperature, and chlorophyll content, positively correlate with water stress [14]. These changes ultimately cause a reduction in such physiological processes as stomatal conductance, transpiration, and photosynthesis [13,15]. Reduced performance of these physiological parameters results due to moderate to severe water stress during cane growth and development [16]. Reduced relative water content, more negative leaf osmotic potential, and loss in turgor potential are the chief restrictions reflected in cane growth for resistance against water deficit [17,18,19]. Free proline accumulation in the leaves and roots is a response of the plant to avoid cell–level injuries due to severe drought by assisting and stabilizing the membrane, making osmotic adjustments and scavenging oxygen radicals [16]. 

Water deficit is a very common global issue, and it is getting harsher daily [20,21,22,23]. The hydrophilic association of water with sugarcane and the dependence of many traits on water availability demands research on identifying a smart number of responsible traits for developing drought–tolerant cane varieties [24]. The screening of water stress–tolerant cane clones remains the aim of many sugarcane genomic improvement programs, which require imperative physiological trait identification as principles for the assortment [1,25]. Such determined physiological and agronomic traits for sugarcane crops can further be used for the screening and development of new germplasm under biotic and abiotic stressors [1,13,26,27]. The drought and water deficit effect is much pronounced on millions of hectares of land, with special reference to sugarcane cultivation. Identifying and screening available germplasm is a valuable target to assure cane yields, even with reduced water consumption and irrigation frequency [3,27]. Physiological traits identified for screening the sugarcane clones under water deficit conditions were utilized in this study to assess the response of the locally available cane germplasm for its performance under sandy loam texture soils. The aim of the study was to identify cane clones for water–limited conditions through agronomic and physiological approaches, which are potentially applicable to water–scarce regions of Pakistan. These drought–resistant, drought–tolerant, and drought–associated physiological traits were analyzed for screening suitable cane clones based on their agronomic and physiological performance under water–stressed conditions. 

## 2. Results 

Two years of statistical analysis showed significant variation in all studied traits comprising cane length and girth, photosynthetic rate, transpiration rate, gas stomatal conductance, proline content, sugarcane yield, leaf temperature, relative water content, water use efficiency, and osmotic potential (Table 1). During 2021–2022, the interaction effect for leaf temperature was found insignificant (*p* ≥ 0.05). 

### 2.1. Yield Traits

**Figure 1:**
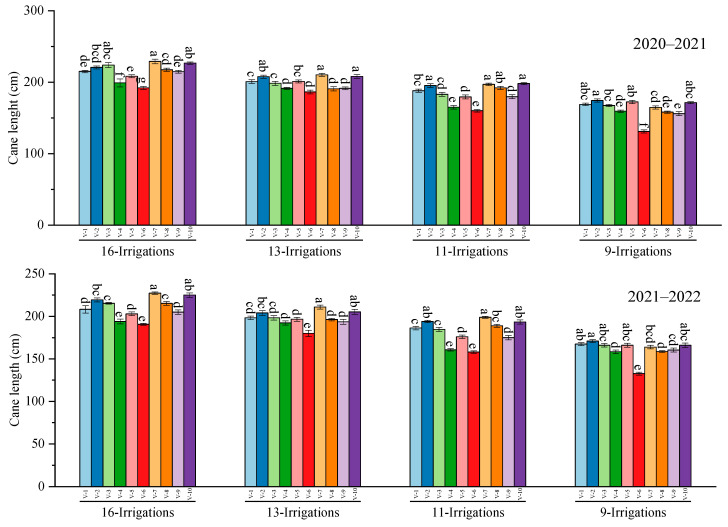

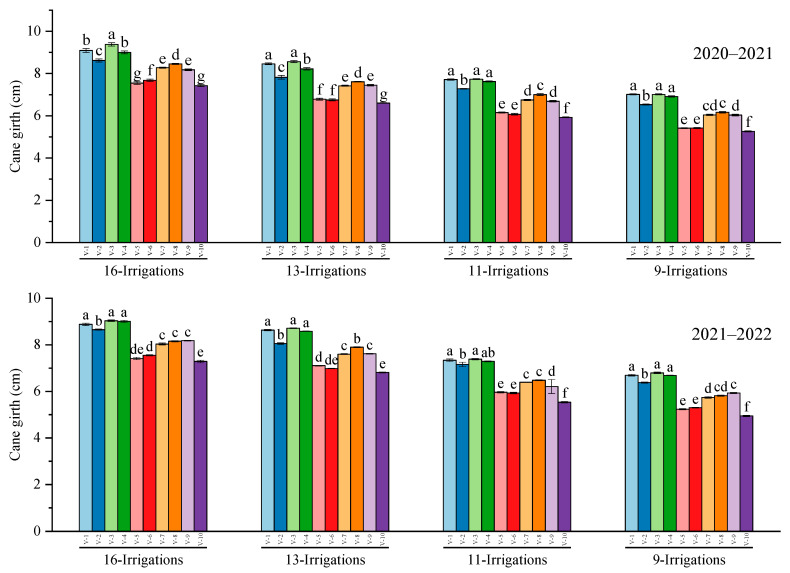
Performance of 10 cane varieties for cane length and girth under different water deficit levels over the years. V1 = HSF–240, V2 = SPF–213, V3 = CPF–249, V4 = CP 77–400, V5 = S2008–FD–19, V6 = S2006–US–469, V7 = S2007–AUS–384, V8 = S2003–US–633, V9 = S2003–US–127, and V10 = S2006–US–658; Variations in lowercase show significant difference between water deficit levels over the years at the *p* ≤ 0.05.

Results regarding cane length exposed significant (*p* ≤ 0.05) responses for different genotypes under varying irrigation regimes. However, the highest cane length was attained by the S2007–AUS–384 sugarcane genotype, whereas the lowest cane length was recorded in CP 77–400 genotypes that were statistically similar to the S2008–FD–19 genotype during both years of study under all irrigation regimes. For cane girth, the maximum cane girth was measured in HSF–240, statistically similar to the CPF–249 sugarcane genotype. Moreover, the minimum cane girth was exposed in S2006–US–658, followed by CP 77–400 and S2008–FD–19 under all irrigation levels (Figure 1).

**Figure 2:**
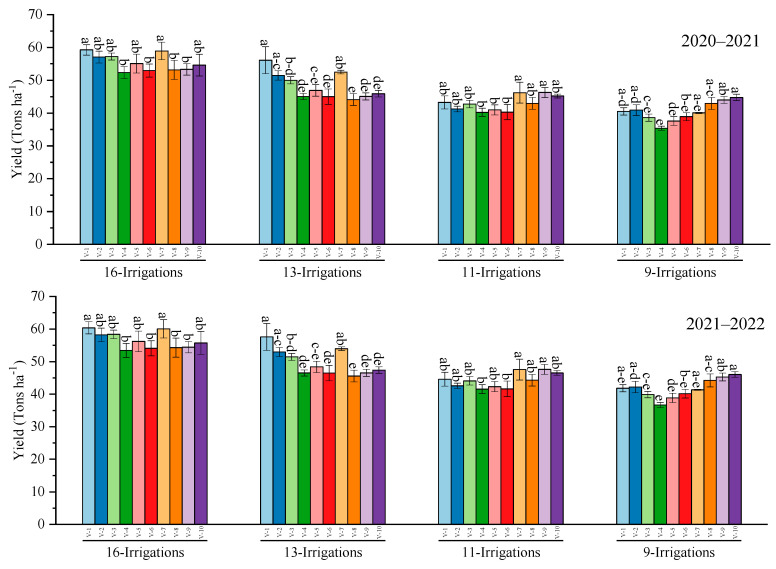
Performance of 10 cane varieties for cane yield under different water deficit levels over the years. V1 = HSF–240, V2 = SPF–213, V3 = CPF–249, V4 = CP 77–400, V5 = S2008–FD–19, V6 = S2006–US–469, V7 = S2007–AUS–384, V8 = S2003–US–633, V9 = S2003–US–127, and V10 = S2006–US–658. Variations in lowercase show significant difference between water deficit levels over the years at the *p* ≤ 0.05.

Similarly, the highest sugarcane yield was recorded in HSF–240 and S2007–AUS–384 among all genotypes under all water deficit levels during both years, whereas the lowest cane yield was collected in CP 77–400 when 13 and 9 irrigations were applied in both years (Figure 2).

### 2.2. Photosynthetic and Transpiration Rate

**Figure 3:**
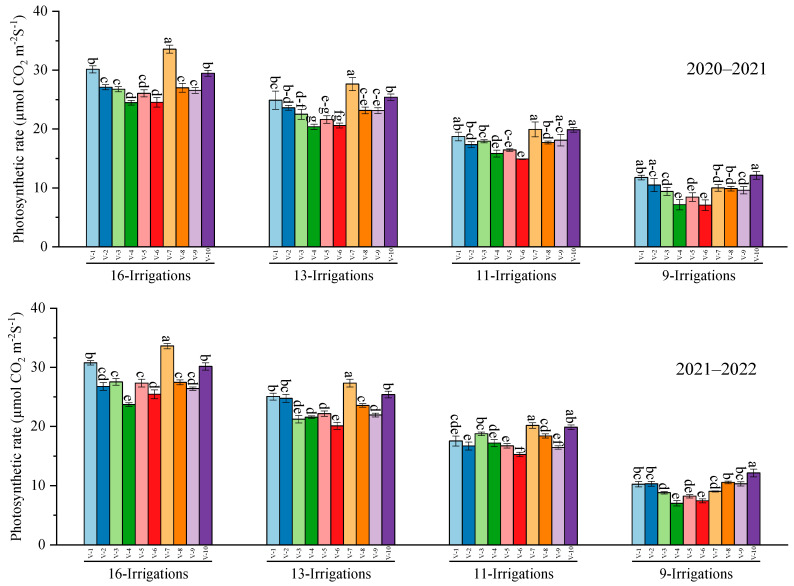

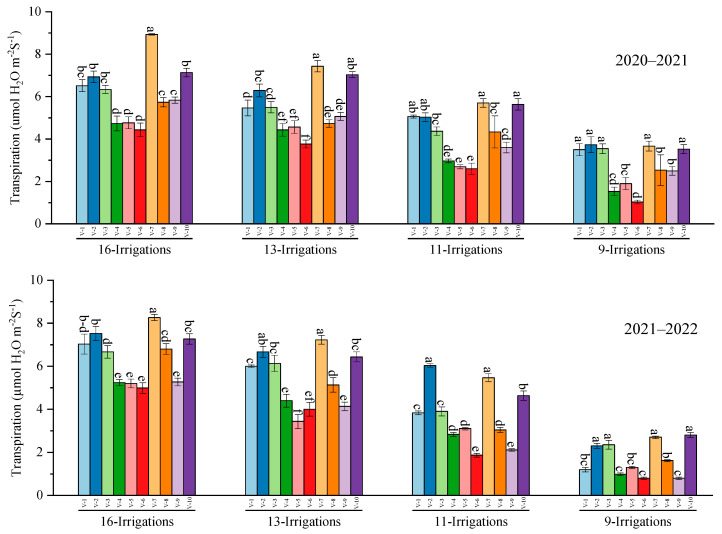
Performance of 10 cane varieties for photosynthetic and transpiration rate under different water deficit levels over the years. V1 = HSF–240, V2 = SPF–213, V3 = CPF–249, V4 = CP 77–400, V5 = S2008–FD–19, V6 = S2006–US–469, V7 = S2007–AUS–384, V8 = S2003–US–633, V9 = S2003–US–127, and V10 = S2006–US–658. Variations in lowercase show significant difference between water deficit levels over the years at the *p* ≤ 0.05.

Data regarding net photosynthetic rate highlighted similar trends during 2020–2021 and 2021–2022 for different genotypes under differential irrigation levels. Results showed that the highest net photosynthetic rate was observed in S2006–US–658, statistically at parity with S2007–AUS–384, and the lowest rate was exhibited by CP 77–400, followed by S2006–US–469 and S2008–FD–19 under different irrigation regimes. Regarding transpiration rate, the maximum value was recorded in S2007–AUS–384, statistically at par with S2006–US–658, whereas the minimum transpiration was observed in CP–77400 during 2020–2021. During 2021–2022, the highest transpiration rate was recorded in S2007–AUS–384, at parity with SPF–213 and S2006–US–658, and the lowest transpiration rate was assimilated in CP–77400 under different irrigation regimes (Figure 3).

### 2.3. Description of Stomatal Conductance and Proline

**Figure 4:**
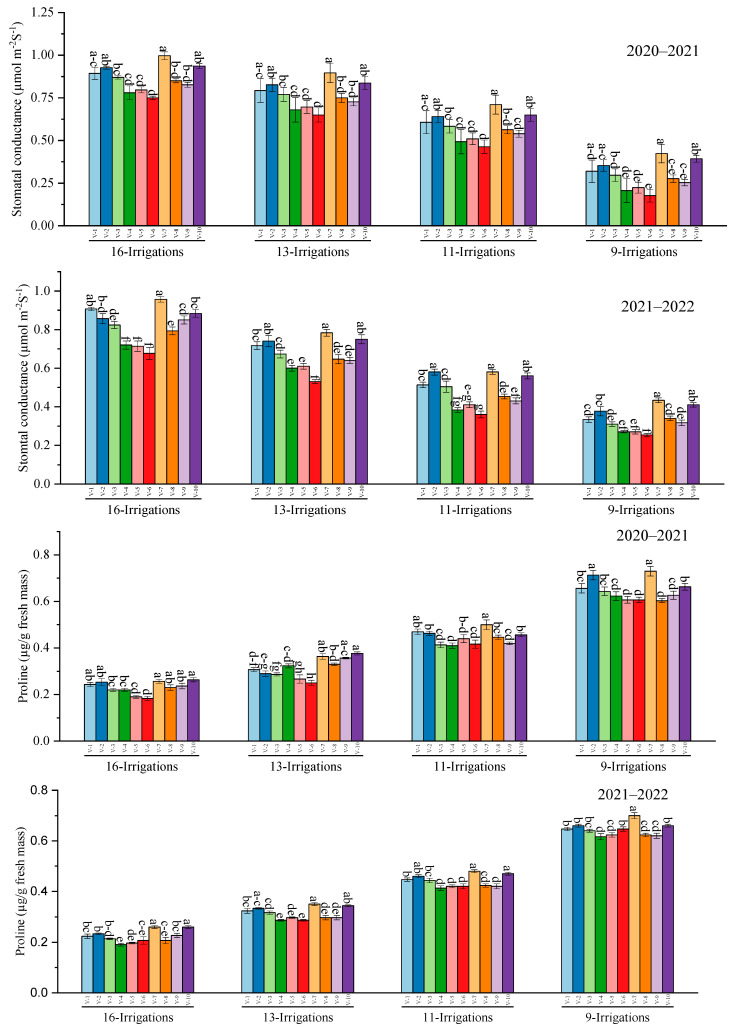
Performance of ten cane varieties for stomatal conductance and proline content under different water deficit levels over the years. V1 = HSF–240, V2 = SPF–213, V3 = CPF–249, V4 = CP 77–400, V5 = S2008–FD–19, V6 = S2006–US–469, V7 = S2007–AUS–384, V8 = S2003–US–633, V9 = S2003–US–127, and V10 = S2006–US–658. Variations in lowercase show significant difference between water deficit levels over the years at the *p* ≤ 0.05.

Stomatal conductance data showed that S2007–AUS–384 and SPF–213 were the leading sugarcane genotypes, whereas the S2006–US–469 was the lowest performer under different irrigation regimes during 2020–2021. For 2021–2022, the highest gas stomatal conductance was measured in S2007–AUS–384, and the lowest was recorded in CP 77–400 under different irrigation levels. Moreover, concerning results regarding proline content, the highest was assessed in the S2007–AUS–384 genotype under 9 irrigation treatments during both years, which was statistically insignificant with SPF–213. In contrast, the lowest proline content was accumulated in S–2006–US–469 and CP 77–400 genotypes under all irrigation regimes during 2020–2021 and 2021–2022 (Figure 4).

### 2.4. Leaf Temperature and Osmotic Potential

**Figure 5:**
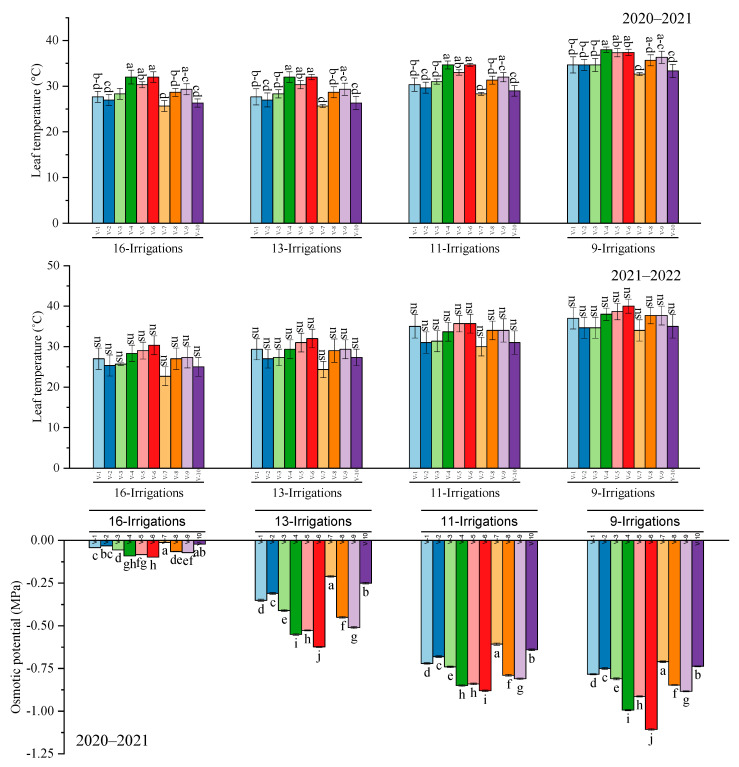

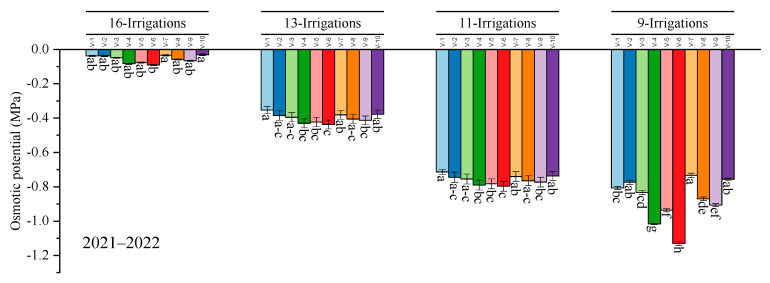
Performance of 10 cane varieties for leaf temperature and osmotic potential under different water deficit levels over the years. V1 = HSF–240, V2 = SPF–213, V3 = CPF–249, V4 = CP 77–400, V5 = S2008–FD–19, V6 = S2006–US–469, V7 = S2007–AUS–384, V8 = S2003–US–633, V9 = S2003–US–127, and V10 = S2006–US–658.

Results regarding leaf temperature exhibited a significant response (*p* ≤ 0.05) towards sugarcane yield and irrigation regime, and the maximum leaf temperature resulted in CP 77–400, followed by S2006–US–469 and S2008–FD–19. The minimum leaf temperature was recorded in HSF–240 and S2006–US–658 under all irrigation regimes in 2020–2021, whereas leaf temperature during 2021–2022 resulted in a non–significant variation. Significantly, the highest osmotic potential was determined in S2007–AUS–384, whereas the lowest potential was assessed in S2006–US–469 under all irrigation regimes during 2020–2021. Similarly, in 2021–2022, the maximum osmotic potential was recorded in HSF–240 sugarcane genotypes where 11 and 13 irrigations were applied, whereas under 9 and 16 irrigations, S2007–AUS–384 and S2006–US–658 had the highest osmotic potential, respectively. However, the least osmotic potential was assessed in S2006–US–469 and CP 77–400 by employing 9 and 16 irrigations, respectively (Figure 5).

### 2.5. Relative Water Content and Water Use Efficiency

**Figure 6:**
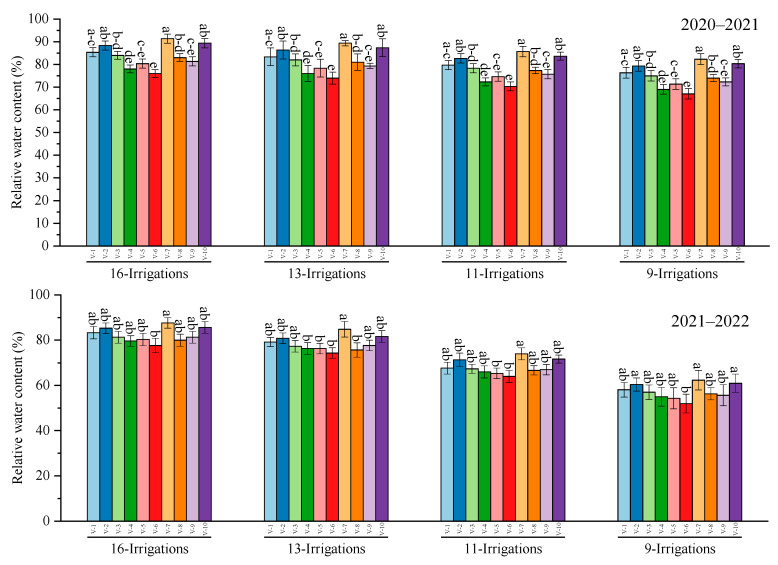

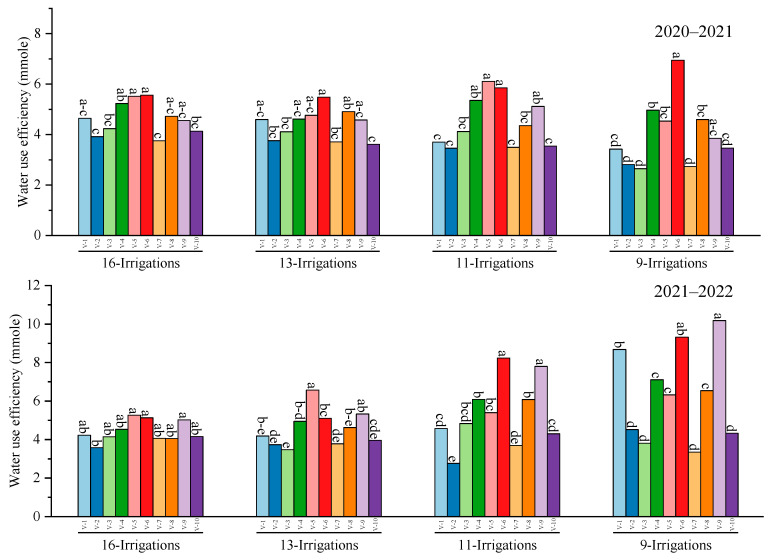
Performance of 10 cane varieties for relative water content and water use efficiency under different water deficit levels over the years. V1 = HSF–240, V2 = SPF–213, V3 = CPF–249, V4 = CP 77–400, V5 = S2008–FD–19, V6 = S2006–US–469, V7 = S2007–AUS–384, V8 = S2003–US–633, V9 = S2003–US–127, and V10 = S2006–US–658.

Data determined the highest relative water content was in S2007–AUS–384 and S2006–US–658 under all irrigation regimes during 2020–2021, while the minimum relative water content was calculated in S–2006–US–469, statistically on par with CP 77–400 and S2008–FD–19 in all irrigation regimes. The overall more relative water content was measured during 2021–2022. In the case of water use efficiency, the highest water use efficiency was calculated in S2006–US–469, similar to CP 77–400 and S2008–FD–19, whereas the least water use efficiency was recorded in SPF–13 under all irrigation treatments during 2020–2021. However, during 2021–2022, the highest water use and water use efficiency was determined in S2003–US–127 under 9 irrigations, and the least value was observed in SPF–213 through employing 11 irrigations. (Figure 6). 

### 2.6. Pearson Correlation Analysis of Physiological and Agronomic Traits of Sugarcane

**Figure 7:**
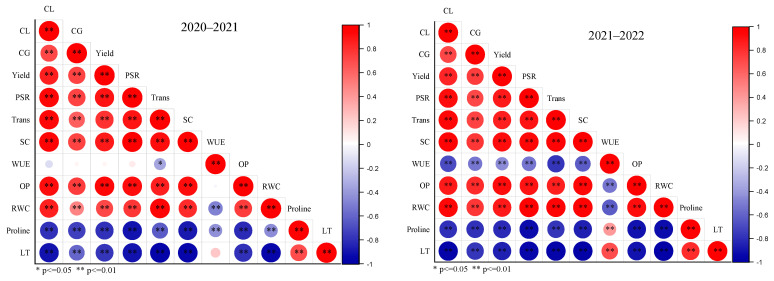
Pearson correlation analysis of studied traits: CL = cane length, CG = cane girth, PSR = photosynthetic rate, Trans = Transpiration, SC = stomatal conductance, WUE = water use efficiency, OP = osmotic potential, *RWC* = relative water content, proline, and LT = leaf temperature of sugarcane cultivars under differential irrigation levels.

Two years (2020–2021 & 2021–2022) correlation analysis assessed the relationship of studied traits (Figure 7). During 2020–2021, sugarcane yield positively correlated with cane girth (CG) and cane length (CL), thus showing these traits had positively contributed to cane yield. Moreover, stomatal conductance (SC), transpiration (Trans), and photosynthetic rate (PSR) also positively correlated with CG, CL, and yield; hence these traits significantly influenced the cane yield. Osmotic potential (OP) and relative water content (*RWC*) showed a strong positive correlation with CL, CG, Yield, PSR, Trans, and SC traits. Similarly, proline and leaf temperature (LT) showed a strong negative correlation with all studied traits, excluding water use efficiency (WUE), but LT showed a positive correlation with proline content. However, 2021–2022 data also showed a similar correlation trend, except for WUE, which negatively correlated with CL, CG, yield, PSR, Trans, and SC.

### 2.7. Principal Component Analysis Description

**Figure 8:**
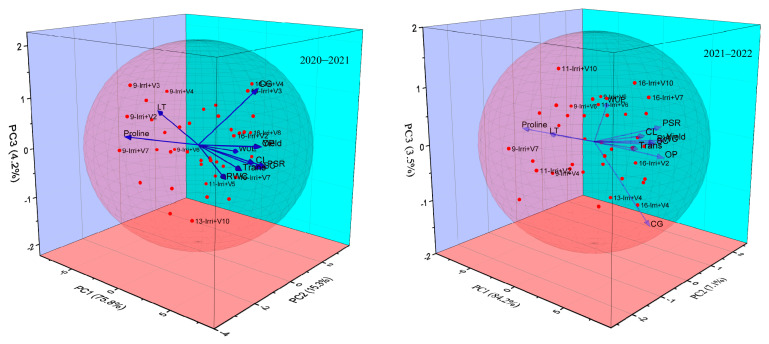
Principal component analysis of targeted sugarcane traits of different genotypes under differential irrigation regimes over the years.

Principal component analysis was presented in 3–D plots during both years (2020–2021 and 2021–2022) of the study (Figure 8). Two years of analyses exhibited that PC–1 had shown the highest variation, followed by PC–2 and PC–3. In 2020–2021, cane length (CL), photosynthetic rate (PSR), transpiration (Trans), and stomatal conductance (SC) were closely related. In addition, overall relative water content (*RWC*) was the maximum in treatment 11–Irri+V5 (11 Irrigations + V5 = S2008–FD–19), whereas transpiration was the highest in 16–Irri+V7 (16–Irirgations + V7 = S2007–AUS–384). Moreover, the highest cane girth (CG) was exhibited in 16–Irri+V4 & 16–Irri+V3 (16 Irrigations + V4 = CP 77–400 & V3 = CPF–249). Treatment 9–Irri + V7 had greater content of proline whereas, 9–Irri+V2 (9–Irrigations + V2 = SPF–213 & V3 = CPF–249) showed the maximum value of leaf temperature. Correspondingly, in the 2021–2022 study PSR, yield, SC, and CL are closely related, and similar findings were also recorded during this year. In this year, the highest water use efficiency was calculated in 9–Irri + V9 (9–irrigations + V9 = S2003–US–127).

## 3. Discussion

Low water availability has drastically impacted cane clones and reduced the growth and development of cane plantations. Similar findings were observed as a result of this experiment when considered based on the physiological traits of these cane clones. Cane clones analyzed through Biplot analysis underwent screening for water deficit tolerance using physiological markers. Water scarcity causes a retardation of plant growth. Plant growth decline during drought occurs due to changes in plant water relationships, reduced assimilation of CO_2_, cellular oxidative stress, damage to membranes in affected tissues, and inhibition of enzyme activities [1].

Results confirmed significantly better growth and development of cane clones being grown under normal irrigation when compared with water–stressed conditions during both experimental years, as described by Silva et al. [13], who opined similarly regarding the productivity and physiology of sugarcane under sandy loam soil conditions. The variation in cane growth response to drought stress is contingent upon the genetic makeup of sugarcane genotypes [28,29]. Some clones were typically screened for their better performance under normal irrigation regimes, while others were found to be differentially better performers in moderate and severe water deficit conditions. Under sandy loam soils, the regenerative ability of cane was conspicuously observed, showing an immediately triggered growth response of clones to water availability in irrigation spells. While some of the clones were weak responders to water availability following water deficit exposures. Such clones having regenerative abilities were S2006–US–658, S2007–AUS–384, and HSF–240, whereas clone names were weak responders, as mentioned above. Similarly, cane clones group CP 77–400, S2008–FD–19, S2006–US–469, and S2003–US–633 showed a medium response or were insensitive to water deficit levels in sandy loam soil for physiological traits. On the other hand, cane clones S2003–US–127, CPF–249, and SPF–213 had better performance for photosynthesis rate, transpiration rate, stomatal conductance, chlorophyll content, osmotic potential, relative water content, proline content, and leaf temperature traits had high values under moderate and severe water deficit levels.

The varieties S2007–AUS–384 and S2006–US–658 were suitable under control and water deficit levels (D1) for those sandy loam texture soils during year I and year II. These clones show non–significant (*p* ≥ 0.05) differences in their performance under mild water deficit compared to control in both years. Thus, clones may be successfully cultivated without any decline in sugarcane production with judicial use of water in sandy loam conditions. Similarly, clones S2003–US–127 and CPF–249 could be grown under medium to severe water deficit conditions, having higher production and survivability in sandy loam soil texture during years I and II. During years I and II, cane clones S2007–AUS–384, S2006–US–658, and SPF–213 had the best performance in control conditions due to good water availability, and these clones produced the highest biomass production under control conditions. Still, their performance reduced significantly due to decreased water quantity from mild, moderate, and severe stress conditions in a sandy loam environment. Cane clones S2003–US–127, CPF–249, and SPF–213 were retained and resistant to drought spells and had maximum values of physiology markers in moderate and severe water stress and sandy loam soil conditions. The performance of these cane clones during year II significantly differs from year I. 

In the study, these cane clones were screened out for high rates of physiological mechanisms under drought spell duration of the crop growth and development in sandy loam soil conditions. The high water demand is a great concern for the sugarcane crop production system among agriculture policymakers, who often suggest substituting the sugarcane crop with other sugar crops that demand less irrigational water [30]. Therefore, the reduction in water mandate of the cane clones is an important part of the sustainable sugarcane crop system. The leakage of electrolytes from leaf samples is associated with membrane damage resulting from oxidative stress [31,32]. The oxidative stress induces injury to the cell membrane, which allows for the diffusion of electrolytes and ions. Under drought stress conditions, the destruction of thylakoid membranes directly or indirectly impacts the chlorophyll content [33]. Drought causes a decline in the SPAD value of sugarcane leaves after exposure to drought [9,34,35]. SPAD value was employed in screening drought tolerance in sugarcane, and under drought stress, it could be used for screening for drought–tolerant and drought–sensitive genotypes [3]. Soil texture also profoundly affected sugarcane productivity, and higher production could be obtained in sandy loam soil. Cane clones S2003–US–127, CPF–249, and SPF–213 had better performance under sandy soil and thus may be suitable for the specific sandy loam soil types. Based on studied cane clones, S2003–US–127, CPF–249, and SPF–213 had high production in most of the cane traits during year II, followed by agronomic intervention in a drought period of crop growth under sandy loam soil conditions in moderate (D2) and severe (D3) stress levels as compared to year I. Sandy loam soil had less water holding capacity and other media necessary for growth and development, the enactment of cane clones S2003–US–127, CPF–249, and SPF–213 proved that under drained water conditions during both years. So, cane clones S2003–US–127, CPF–249, and SPF–213 performed better regarding agronomic and physiological assessment under moderate to severe drought stress situations in sandy loam soil conditions. Drought has a significant impact on photosynthesis, primarily through the substantial decrease in stomatal conductance resulting from stomatal closure in response to drought [33]. The reduction in stomatal conductance, caused by drought stress, can adversely affect various photosynthetic processes. Several studies on sugarcane have reported that varieties considered more sensitive to drought stress exhibit greater stomatal closure and reduced transpiration [26,34]. In our study rate of photosynthesis, transpiration rate, and stomatal conductance were decreased after inducing water stress. These findings are similar to those of Medeiros et al. [16] and Natarajan et al. [36]. Numerous studies have demonstrated the significant impact of drought on photosynthetic traits, including the net photosynthetic rate. Drought conditions often coincide with low soil moisture and high air temperature, and this combination of factors leads to increased evapotranspiration and subsequently affects photosynthesis in field conditions da Graca et al., [37]. Additionally, when drought stress was imposed, it was observed that drought–sensitive sugarcane varieties experienced a significant decrease in transpiration rate. In certain sugarcane genotypes, however, the plants could sustain their photosynthetic activity and maintain their water status by developing deeper roots [38]. 

Water use efficiency is a crucial characteristic when selecting drought–resistant varieties [39]. Drought–tolerant sugarcane genotypes exhibit higher intrinsic instantaneous water use efficiency, along with the ability to maintain higher water potential and photosynthetic capacity during water deficit conditions [40]. Drought stress has a substantial impact on sugarcane yield. Water deficit stress results in stunted growth and limited tillering, leading to empty and low–quality millable stalks. Consequently, exposure to drought stress during the early growth stages and midseason leads to reductions in both cane and sugar yields [28,29]. Hemaprabha [41] reported that water stress treatments had a negative impact on cane yield and total dry weight when compared to irrigation treatments. Insufficient water availability during the formative phase resulted in significant changes in yield and its related parameters. Specifically, after withholding water for 90 days during the formative phase, there were reductions in single stalk weight, cane height, and internode length. Under prolonged drought stress conditions, there was a considerable reduction in cane yield of approximately 21%. Furthermore, significant variations were observed among different genotypes for nearly all the traits evaluated, except for stalk length and diameter, when subjected to moderate drought stress conditions [42]. 

The approach of either spending or conserving water depends on factors such as the timing, intensity of the drought period, and the specific sugarcane genotype in a given location. The findings indicate that maintaining optimal photosynthetic performance during drought and facilitating recovery after re–watering, particularly during the stalk growth stage, play a vital role in determining the final yield of sugarcane. In the future, climate–smart breeding combined with conventional breeding strategies can be employed to develop climate–resilient sugarcane cultivars [43,44,45,46,47].

## 4. Materials and Methods

### 4.1. Field Preparation

Optimum land preparation is an important segment regarding the physical anchorage of the plant, which help the plant to uptake soil water and nutrition. Better tillage operation for the planting of crops is good practice concerning water holding capacity as well as infiltration rate in the case of conservation agriculture; hence 500 m^2^ sandy loam soil was selected at Ramzan Sugar Mills, Limited, for the experiment. For preparation, firstly a disc harrow was used to open the soil, and two ploughings were done with the help of a cultivator. After a good tilth, the trenches were made with the help of a sugarcane planter, the trenches being 120 cm wide. Soil sampling was done at 0–30 cm depth of soil by using soil auger and composite soil samples were analyzed by using standard procedures and protocol and analysis that is given in supplementary data (Table S1, Figure S1) as well as weather data of subject study is given in supplementary data.

### 4.2. Collection of Planting Material and Trial Execution

For experimentation, ten cane genotypes were collected from different Ramzan Sugar Mill farms, which are mentioned above. The proposed study was conducted at the University of Agriculture, Faisalabad, during spring 2020–2021. In both years, a fresh crop was planted. Experiments were replicated three times with four drought levels, i.e., 16 irrigations, 13 irrigations, 11 irrigations, and 9 irrigations upheld via alternate irrigation methods in sandy loam soil. Sugarcane varieties S2003–US–127, CP 77–400, SPF–213, S2006–US–469, CPF–249, S2003–US–633, S2006–US–658, S2007–AUS–384, S2008–FD–19, and HSF–240 were sown in 120 cm wide trenches with a net plot size of 2.4 m × 6.0 m and double budded cane setts were planted through hand placement in single row at 7500 setts ha^−1^ during spring 2021 and 2022. Recommended macronutrient–based fertilizer doses (NPK 168:112:112 kg ha^−1^) and plant protection measures were adopted to ensure uniform growth. The experiment was laid out in a randomized complete block design (RCBD) with a split–plot arrangement. The crop was irrigated by imposing water deficit levels, i.e., D_0_ (16 irrigations), D_1_ (13 irrigations), D_2_ (11 irrigations), and D_3_ (9 irrigations). 

### 4.3. Data Collection

Physiological traits were recorded at the tillering and grand growth phases of cane clones by using an infra–red gas analyzer (IRGA) for the determination of stomatal conductance, gaseous exchange, photosynthesis rate, and transpiration rate between 9:00 a.m. and 11:00 a.m. [48]. Chlorophyll content was recorded using SPAD model MC–100, Apogee Pvt. Leaf osmotic potential was also estimated using an osmometer model VAPRO, while agronomic traits, such as cane length and girth, were recorded at harvest maturity.

To determine relative water content, leaf disks were collected with a 2 mm diameter cork borer from the same leaf samples frozen on ice in a glass vessel. The fresh weight (*W_f_*) of leaf disks was measured within 2 h of separation, which were then saturated in water, and reweighed after four hours to get their turgid/ saturated weight (*W_t_*) at room temperature. Leaf discs were rapidly stained dry and oven dried for 48 h at 80 °C for measuring dry weight (*W_d_*). *RWC* was calculated from the following equation [49]:

$$RWC=\frac{\left(W_{f}-W_{d}\right)}{\left(W_{t}-W_{d}\right)}\times 100$$

Proline content was determined in the normal and stressed cane leaves using the procedure described by Bates et al. [50]. Moreover, leaf temperature was recorded by using a hand–held infrared thermometer [13]. 

### 4.4. Statistical Analysis

Two years of experimentation were executed using a randomized complete block under split plot arrangements, and collected data were analyzed using OriginPro 2022 software. Treatment means were presented using paired comparison plot techniques, and the LSD test was employed to differentiate means at 5% probability. However, Pearson correlation analysis was done by deploying a two–tailed *t*–test (df–2). 

## 5. Conclusions

Regardless of the complex interactions of physiological parameters in cane genetics, drought, and environmental effects during year I and year II, it was concluded that S2006–US–658, S2007–AUS–384, and HSF–240 sugarcane genotypes had better resistance to water deficit and low water availability. However, there is still a need to assess the lowest water requirement that will help these clones grow sustainably. Therefore, the aforementioned cane genotypes are screened out for drought conditions, and S2006–US–658, S2007–AUS–384, and HSF–240 for irrigated conditions under the sandy loam texture.

## Figures and Tables

**Table 1:** F value of following traits of sugarcane genotypes under varying irrigation levels.

Traits	2020–2021			2021–2022		
	I	G	I × G	I	G	I × G
Cane length	406.84 **	96.9 **	7.43 **	833.64 **	100.17 **	6.1 **
Cane girth	4074.89 **	1078.04 **	2.08 **	4509.79 **	490.87 **	2.37 **
Photosynthetic rate	315.8 **	44.95 **	2.56 **	224.4 **	5.36 **	1.27 *
Transpiration rate	213.66 **	74.39 **	2.12 **	1196.16 **	112.38 **	5.13 **
Gas Stomatal conductance	227.84 **	55.64 **	0.05 *	913.81 **	73.92 **	2.12 **
Proline content	3873.95 **	22.43 **	3.73 **	8653.61 **	46.36 **	13.37 *
Yield	24.73 **	8.93 **	3.2 **	19.95 **	8.93 **	3.2 **
Leaf temperature	46.03 **	36.34 **	0.22 ^ns^	18.3 **	5.53 **	0.08 ^ns^
Relative water content	13.95 **	30.75 **	0.08 *	35.94 **	7.2 **	0.05 *
Water use efficiency	9.66 **	13.64 **	1.16 *	136.18 **	23.3 **	4.71 *
Osmotic Potential	36,267.3 **	2251.26 **	166.01 *	476.64 **	185.9 **	52.69 **

*, **, significant at *p* ≤ 0.05, *p* ≤ 0.01, respectively.

## Data Availability

All data generated or analyzed during this study are included in this published article.

## Supplementary Materials

The following supporting information can be downloaded at: https://www.mdpi.com/article/10.3390/plants12152759/s1, Figure S1: Weather data during the course of study of 2020–2021 and 2021–2022; Table S1: Soil profile analysis of 2020–2021 and 2021–2022.
