# Supplementary material for: Comparative Analysis of Growth and Physiological Responses of Sugarcane Elite Genotypes to Water Stress and Sandy Loam Soils
Source: Plants (Basel). 2023 Jul 25;12(15):2759. doi: 10.3390/plants12152759 (PMC10421443; doi:10.3390/plants12152759)
Supplement: Supplementary file 1 [file plants-12-02759-s001.zip › plants-2476627-supplementary.pdf]

Table. Soil profile analysis of 2020-21 and 2021-22.

Soil Texture	pH	EC	N	P	K	O.M	Saturation %	Moisture contents	Ca+Mg	Na^+	Cl^-
		2020-21									
		dSm^{-2}	%	ppm	ppm	%	%	%	meL^{-1}	meL^{-1}	meL^{-1}
Sandy loam	7.5	1.95	0.344	0.85	39	0.07	17	22	15	4.5	4
		2021-22									
	7.6	1.86	0.36	0.89	36	0.06	16	21	16.1	4.87	4.1

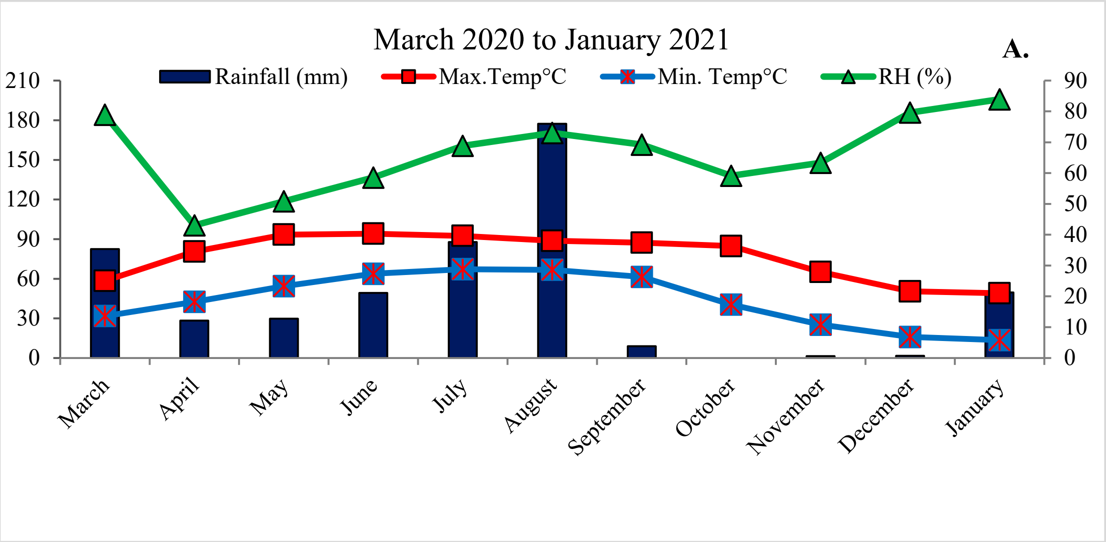

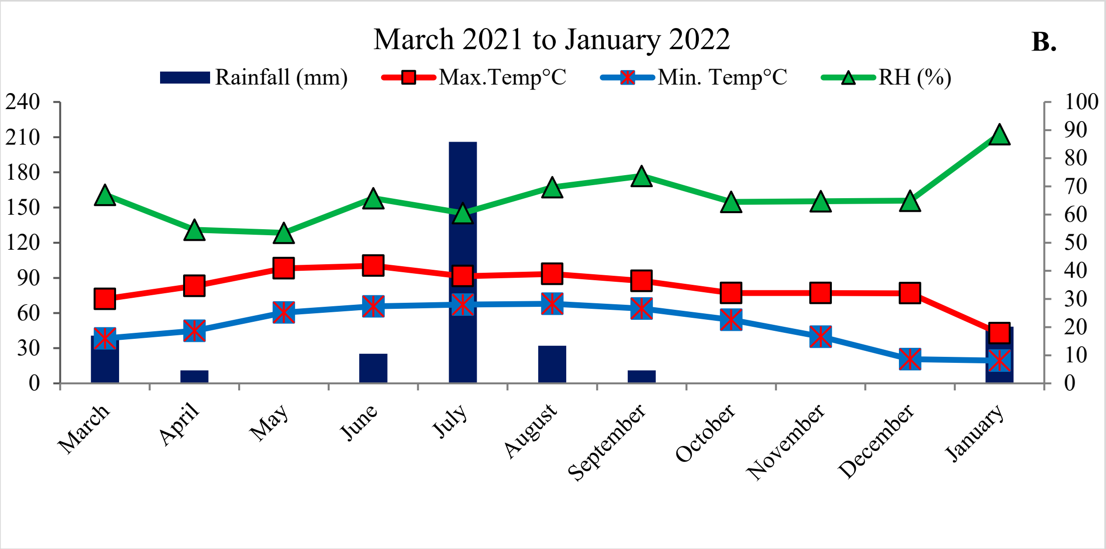

Figure. Weather data during the course of study of 2020-21 and 2021-22.
